# Tumor infiltrating neutrophil might play a major role in predicting the clinical outcome of breast cancer patients treated with neoadjuvant chemotherapy

**DOI:** 10.1186/s12885-021-07789-6

**Published:** 2021-01-14

**Authors:** Sheng-Kai Geng, Shao-Mei Fu, Shi-Hong Ma, Yi-Peng Fu, Hong-Wei Zhang

**Affiliations:** 1 Department of General Surgery, Xuhui District Central Hospital of Shanghai, Shanghai, 200031 China; 2 Department of Breast Surgeon, The Obstetrics & Gynecology Hospital of Fudan University, Shanghai, 200011 China; 3 Department of General Surgery, Zhongshan Hospital, Fudan University, Shanghai, 200032 China

**Keywords:** Breast cancer, Tumor infiltrating neutrophil, Chemotherapy efficiency, Disease-free survival, Neoadjuvant chemotherapy

## Abstract

**Background:**

This study was aimed to explore the predictive ability of tumor infiltrating neutrophil (TIN) in patients with breast cancer treated with neoadjuvant chemotherapy (NACT). Furthermore, the significance of TIN’s dynamic change before and after NACT was investigated.

**Methods:**

Between January 2004 and December 2017, a total of 133 patients with breast cancer who underwent NACT before surgery were enrolled in this retrospective cohort. Eighty-nine of them were able to get the core needle biopsy (CNB) samples and all the pathological samples after surgery were available. TIN was detected by immunohistochemical staining of CD66b. The optimal cut-off value was determined via receiver operating characteristic (ROC) curve analysis. The association of clinicopathologic characteristics and chemotherapy efficiency was analyzed using X^2^ test or Fisher’s exact test or t-test as appropriate, and the prognostic significances were assessed by univariate and multivariate analyses.

**Results:**

Patients with higher TIN after NACT were confirmed to be significantly associated with worse prognosis (*P* = 0.002). After stratifying patients into two groups, high difference group was prone to have better chemotherapy efficiency (*P* < 0.001) and clinical outcome in both univariate (*P* = 0.002) and multivariate analyses (*P* = 0.003).

**Conclusions:**

In this study, higher TIN after NACT was confirmed to be associated with breast cancer patients’ worse chemotherapy efficiency and shorter disease-free survival (DFS). Furthermore, the TIN’s dynamic change before and after NACT was firstly proved to be a more accurate predictive marker compared with TIN after NACT.

**Supplementary Information:**

The online version contains supplementary material available at 10.1186/s12885-021-07789-6.

## Background

Breast cancer is the leading cause of cancer-related deaths in women worldwide [1]. With the enhancement of health awareness and improvement of detection equipment, the incidence of breast cancer has significantly increased in young women [2]. Due to the aggressive biological characteristics [3] and aesthetic concerns, neoadjuvant chemotherapy (NACT) has been widely accepted by breast cancer patients to achieve breast-conserving (BCS) and cosmetic surgery. Although some patients could benefit from NACT, easily accessible and reliable clinical markers are needed to stratify the prognosis of this population.

Immune response plays an essential role in chemotherapy-mediated tumor eradication, which is reflected by the tumor infiltration immune cells in the tumor microenvironment (TME) [4–7], such as tumor infiltrating neutrophils (TIN), which have emerged as activators and regulators of tumor immunity in TME [8–12]. For instance, Galdiero et al. [13] revealed that colorectal cancer patients who had higher TIN density showed better response to 5-FU-based chemotherapy. While in other researches, higher TIN was identified as a prognostic marker for poor clinical outcome in some malignant tumors such as renal cell carcinoma [14], and head and squamous cell carcinoma [15]. Wang, Y et al. also observed that neutrophils in tumor parenchyma were an independent factor for poor prognosis in breast cancer patients [16]. The present study revealed that neutrophils induced breast cancer epithelial-mesenchymal transition (EMT) via tissue inhibitor of metalloproteinase-1 (TIMP-1). Reciprocally, breast cancer cells undergoing EMT enhanced neutrophil TIMP-1 secretion by CD90 in a cell-contact manner. Shaul, ME et al. hypothesized that tumor neutrophils play an important role in the recruitment of immunocytes, and in maintaining the balance between the activation and suppression of the immune system in cancer [17]. However, TIN has been rarely reported in breast cancer, and the significance of dynamic changes in TIN before and after NACT in TME remains unknown.

The goal of this study was to evaluate the clinical value of TIN in patients with breast cancer treated with NACT. Furthermore, the significance of dynamic change in TIN before and after NACT was investigated.

## Methods

### Patients

A total of 133 patients with breast cancer who underwent NACT before surgery were enrolled in this study, of which 89 underwent core needle biopsy (CNB) before NACT, and all the pathological samples after surgery were available. This study was approved by the Institutional Review Board of Zhongshan Hospital, Fudan University (B2020–415). The patients were enrolled from the Zhongshan Hospital, Fudan University, between January 2004 and December 2017. The inclusion and exclusion criteria were as follows: all patients were pathologically diagnosed with breast cancer; all patients received radical treatment for breast cancer; all the blood samples were collected within 3 days before operations; all patients underwent NACT before surgery; chemotherapy efficacy of all patients was evaluated after NACT based on response evaluation criteria in solid tumors (RECIST) combined with pathological residual tumor cells; postoperative pathological samples of all patients were stained to count CD66b positive TINs using immunohistochemistry. We used one marker to identify TIN because after careful search, CD66b neutrophils were found to be localized either within the blood vessels or diffusely scattered throughout the tumor. Jensen HK et al. used double staining (CD34 and CD66b) for neutrophils mainly because there may be more vascular tissue in renal cell carcinoma [18], while other studies only used CD66b staining to represent neutrophils because they comprise 96% of the granulocyte population [19–21]. In our study, breast cancer specimens were mainly gland tissue; 89 patients’ CNB samples before NACT were stained to count CD66b positive TINs using immunohistochemistry; all patients had complete records and follow-up data, including baseline characteristics such as sex, age, menopause status, stage, molecular type, and preoperative routine blood test.

### Histopathological evaluation

Chemotherapy efficacy of all patients was evaluated after NACT based on RECIST [22] combined with pathological evaluation of residual tumor cells. Patients were stratified into two groups, remission group and no remission group. The remission group included patients who achieved complete response (CR) and partial response (PR). For pathological evaluation, CR was defined as the complete absence of invasive disease in the breast tumor according to the (NSABP) B-18 protocol [23], and PR was defined as the absence of invasive tumor or only focal residual invasive carcinoma cells in the primary site [24]. The no remission group included patients who achieved stable disease (SD) and progressive disease (PD) [22].

### Immunohistochemistry and evaluation of immunostaining

The immunohistochemical protocols were performed as previously described [19, 20, 25, 26]. We briefly described as follows: Formalin-fixed paraffin-embedded surgical specimens were used for the immunohistochemical analysis. The presence of available tumor was confirmed by hematoxylin and eosin staining. The tissue blocks were sectioned at 2 μm and mounted on glass slides. The sections were dewaxed in xylene and graded alcohols, hydrated, and washed in phosphate-buffered saline (PBS). After the endogenous peroxidase was inhibited using 3% H2O2 for 30 min, the sections were pretreated in a microwave oven (15 min in sodium citrate buffer, pH 6) and then incubated with 10% normal goat serum for 30 min. The mouse antihuman monoclonal anti-CD66b primary antibody (clone G10F5, diluted at 1:200, BD Biosciences) was applied at room temperature for 1.5 h, and washed with PBS. Then, the biotinylated-labeled secondary antibody was applied for 30 min followed by streptavidin peroxidase. Finally, the sections were stained with hematoxylin for 1 min and mounted with an aqueous mounting medium supplied with the kit. Digital pathological section scanner system was used to scan and collect high-definition staining images. To count the number of tumor-infiltrating immune cells, we randomly selected five high-power fields of view (HPF, 200X), and calculated the average as the count. Positive staining was generally brown and located in the cell membrane. The value of TIN pre-NACT, TIN post-NACT and the change of TIN before and after NACT were calculated (the value of TIN’s change equals the number of pre-NACT TIN minus the number of post-NACT TIN). According to the optimal cut-off value of TIN’s change, patients were stratified into the high difference group and the low difference group. The evaluation of CD66b immunostaining was independently conducted by two pathologists who were blinded to the clinical data, and the results were averaged.

### Follow-up

All patients underwent postoperative follow-up every 3 months during the first postoperative year, and every 6 months thereafter. Routine blood test, chest X-ray, tumor markers, breast, liver, gallbladder, pancreas and spleen ultrasonography were performed in every follow-up. Bone-scan was performed every 12 months. The disease free survival (DFS) was defined as the interval between surgery and time of recurrence for relapsed patients or from the date of surgery to the date of last follow-up for nonrecurrent patients.

### Statistical analysis

Statistical analysis was performed using SPSS version 22. The optimal cut-off value of TIN’s change was determined via receiver operating characteristic curve (ROC) curve analysis. Youden index is the sum of the sensitivity and specificity minus 1. We used Youden index to find the optimal cut-off value. The association between clinicopathological characteristics and patients’ clinical outcome was analyzed using X^2^ test or Fisher’s exact test. The Kaplan–Meier analysis was used to compare the survival differences of patients between different cohorts. The Cox proportional hazards regression model was used to perform univariate and multivariate analyses. For all statistical tests, *p* < 0.05 (two-tailed) was considered significant.

## Results

### Clinicopathological profiles of the patients

The clinicopathological characteristics of the patients treated with NACT are presented in Table 1. The median follow-up time was 27 months (range from 10 to 109 months). The median age of all patients was 54.15 ± 10.747 years. Meanwhile, 24 of 133 breast cancer patients experienced recurrences during follow-up. Among all breast cancer patients, 88 were hormone receptor positive (HR +) patients (39 Luminal A patients and 49 Luminal B patients), 17 were human epidermal receptor positive (Her-2 +) patients and 28 were triple negative breast cancer (TNBC) patients. In terms of the chemical regime of patients treated with NACT, 71 patients were treated with docetaxel + anthracyclines + cyclophosphamide (TEC) plans, 23 patients were treated with anthracyclines + cyclophosphamide (EC) plans, 12 were treated with docetaxel + cyclophosphamide (TC) plans, and 27 patients were treated with docetaxel + platinum (DP) plans.

**Table 1:** Clinicopathological profiles of the patients treated with NACT

Characteristic			
**Age**		54.15 ± 10.747	
**< 40**		14	10.5%
**> 40**		119	89.5%
**No menopause**		49	36.8%
**Menopause**		84	63.2%
**T1**		20	15.0%
**T2**		80	60.2%
**T3**		26	19.5%
**T4**		7	5.3%
**N0**		36	27.1%
**N1**		27	20.3%
**N2**		32	24.1%
**N3**		38	28.6%
**Stage I**		9	6.8%
**Stage II**		46	34.6%
**Stage III**		78	58.6%
**HR+**		88	66.2%
**HR-**		45	33.8%
**Her-2 positive**		17	12.8%
**Her-2 negative**		116	87.2%
**TNBC vs**		28	21.1%
**No TNBC**		105	78.9%
**Recurrence VS**		24	18.0%
**No recurrence**		109	82.9%
**CR**	**TEC**	71	53.4%
	**EC**	23	17.3%
	**TC**	12	9.0%
	**TX**	27	20.3%
**Post-NACT neutrophils**		2.92 ± 1.72	
**Post-NACT lymphocyte**		1.30 ± 0.48	

*Abbreviation*: *HR* Hormone receptor, *Her-2* Human epidermal growth factor receptor-2, *TNBC* Triple negative breast cancer, *NACT* Neoadjuvant chemotherapy, *CR* Chemical regime, *TEC* Docetaxel + anthracyclines + cyclophosphamide, *EC* Anthracyclines + cyclophosphamide, *TC* Docetaxel + cyclophosphamide, *TX* Docetaxel + platinum

### Cut-off value of TIN pre-NACT, TIN post-NACT and its dynamic change

CD66b positive staining varied in different specimens of breast cancer patients, and was diffusely scattered throughout the tumor. The value of CD66b positive TIN ranged from 0.2 to 225 (typical staining is shown in Supplement Fig. 3), the typical staining of TIN’s dynamic change is shown in Supplement Fig. 4.

**Fig. 1:**
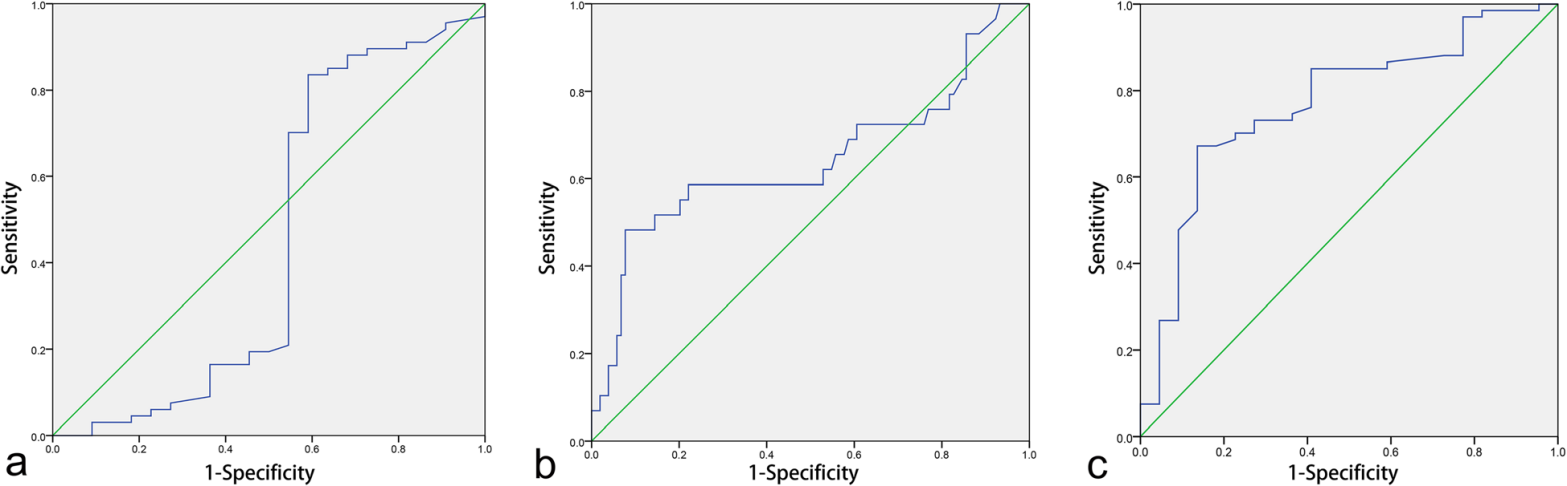
Receiver operating characteristic (ROC) curves of TIN before NACT for chemotherapy efficiency (**a**), TIN after NACT for ineffectiveness of chemotherapy efficiency (**b**) and the difference of TIN before and after NACT for chemotherapy efficiency (**c**). The area under the ROC curve was 0.449 (95% confidence interval [CI], 0.275–0.622, *P* = 0.473), 0.643 (95% confidence interval [CI], 0.509–0.776, *P* = 0.019), 0.770 (95% confidence interval [CI], 0.657–0.883, *P* < 0.001), respectively

In this study, there was no optimal cut-off value of the TIN before NACT for chemotherapy efficacy, and the area under the ROC curve was 0.449 (95% confidence interval [CI], 0.275–0.622, *p* = 0.473, shown in Fig. 1a). The optimal cut-off value of TIN after NACT for resistance to chemotherapy was 52.2, and the area under ROC curve was 0.643 (95% [CI], 0.509–0.776, *p* = 0.019, shown in Fig. 1b). The optimal cut-off value of the TIN’s (CD66b positive staining) change associated with the strongest Youden index for the chemotherapy efficacy was 5.75. The area under the ROC curve was 0.770 (95% [CI], 0.657–0.883, *p* < 0.001, showed in Fig. 1c).

### The value of TIN for chemotherapy efficacy and DFS

**Fig. 2:**
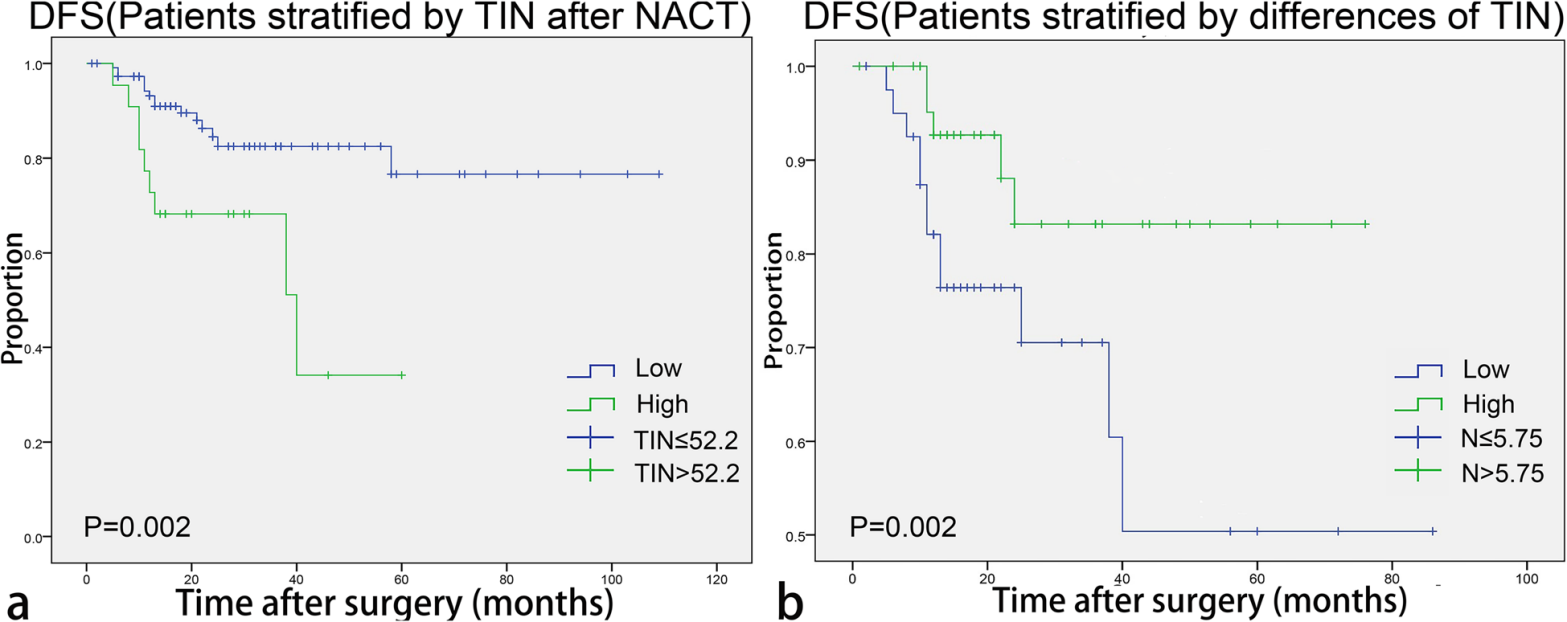
Kaplan-Meier analyses of TIN after NACT for DFS (**a**) and the difference of TIN before and after NACT for DFS (**b**). DFS = disease free survival, TIN = tumor infiltrating neutrophil, NACT = neoadjuvant chemotherapy

Although there was no optimal cut-off value of TIN before NACT for chemotherapy efficacy, the optimal cut-off value of TIN after NACT for resistance to chemotherapy was 52.2. After stratifying patients into two groups, the clinicopathological characteristics of each group are listed in Table 2. Patients with higher TIN were more likely to suffer from resistance after treatment with NACT (*p* < 0.001, shown in Supplement Fig. 2a). In addition, after long term follow-up, higher TIN after NACT was proved to be associated with significantly shorter DFS (*p* = 0.002, hazard ratio [HR] = 3.415; 95% [CI] 1.489–7.831 in univariate analysis, shown in Fig. 2a, and *p* = 0.007, hazard ratio [HR] = 3.265; 95% [CI] 1.374–7.758 in multivariate analysis, shown in Table 3). The prognostic value of TIN’ change was analyzed as follows.

**Table 2:** Clinicopathological profiles of the patients treated with NACT (stratified by TIN after NACT)

Characteristic		Low TIN after NACT	High TIN after NACT	*P*
		*N* = 111	*N* = 22	
**Age**		54.02 ± 10.872	54.82 ± 10.312	0.804
**< 40**		12	2	0.810
**> 40**		99	20	
**No menopause**		42	7	0.593
**Menopause**		69	15	
**T1**		17	3	0.791
**T2**		68	12	
**T3**		21	5	
**T4**		5	2	
**N0**		33	3	0.382
**N1**		21	6	
**N2**		25	7	
**N3**		32	6	
**Stage I**		9	0	0.214
**Stage II**		40	6	
**Stage III**		62	16	
**HR+**		77	11	0.079
**HR-**		34	11	
**Her-2 positive**		11	6	**0.026**
**Her-2 negative**		100	16	
**TNBC vs**		23	5	0.833
**No TNBC**		88	17	
**Remission vs**		96	8	**< 0.001**
**No remission**		15	14	
**Recurrence vs**		15	9	**0.002**
**No recurrence**		96	13	
**CR**	**TEC**	60	11	0.089
	**EC**	20	3	
	**EC**	9	3	
	**TX**	22	5	

*Abbreviation*: *HR* Hormone receptor, *Her-2* Human epidermal growth factor receptor-2, *TNBC* Triple negative breast cancer, *NACT* Neoadjuvant chemotherapy, *CR* Chemical regime, *TEC* Docetaxel + anthracyclines + cyclophosphamide, *EC* Anthracyclines + cyclophosphamide, *TC* Docetaxel + cyclophosphamide, *DP* Docetaxel + platinum

**Table 3:** Clinicopathological Characteristics: Univariate and Multivariate Survival Analyses

Characteristic	DFS			
	Univariate		Multivariate	
	HR (95%CI)	***P***	HR (95%CI)	***P***
**Age**	1.033 (0.995–1.072)	**0.007**	1.031 (0.973–1.093)	0.295
**Menopause vs no menopause**	1.693 (0.672–4.268)	0.257	0.927 (0.227–3.790)	0.916
**Stage (III vs II vs I)**	4.411 (1.396–13.938)	**0.015**	3.801 (1.163–12.427)	**0.027**
**Molecular type (HR+ vs Her-2 vs TNBC)**	0.926 (0.559–1.534)	0.756	0.916 (0.606–1.384)	0.676
**Low TIN after NACT VS High TIN after NACT**	3.415 (1.489–7.831)	**0.002**	3.265 (1.374–7.758)	**0.007**

*Abbreviation*: *HR* Hormone receptor, *Her-2* Human epidermal growth factor receptor-2, *TNBC* Triple negative breast cancer, *DFS* Disease free survival, *TIN* Tumor infiltrating neutrophil, *NACT* Neoadjuvant chemotherapy

### Relationship between the value of TIN’s change and patient characteristics

After confirming the optimal cut-off value of the TIN’s change, 89 breast cancer patients whose pathological specimens were available before and after NACT were classified into two groups, the high difference group (*n* = 48) and the low difference group (*n* = 41). The clinicopathological characteristics of each group are listed in Table 4. There were no significant differences between the value of TIN’s change and menopause status, tumor size, lymph node metastasis, stage and chemical regimes (*p* = 0.495, shown in Supplement Fig. 1).

**Table 4:** Clinicopathological profiles of the paired patients treated with NACT

Characteristic		Low difference value	High difference value	*P*
		*N* = 41	*N* = 48	
**Age**		54.73 ± 11.052	53.06 ± 11.434	0.636
**< 40**		3	9	0.115
**> 40**		38	39	
**No menopause**		15	19	0.772
**Menopause**		26	29	
**T1**		10	6	0.394
**T2**		22	30	
**T3**		7	7	
**T4**		2	5	
**N0**		8	21	0.098
**N1**		9	8	
**N2**		11	7	
**N3**		13	12	
**Stage I**		4	4	0.176
**Stage II**		11	22	
**Stage III**		26	22	
**HR+**		28	32	0.870
**HR-**		13	16	
**Her-2 positive**		4	6	0.683
**Her-2 negative**		37	42	
**TNBC vs**		9	10	0.898
**No TNBC**		32	38	
**Remission vs**		22	45	**< 0.001**
**No remission**		19	3	
**recurrence**		12	5	**0.024**
**No recurrence**		29	43	
**CR**	**TEC**	14	17	0.495
	**EC**	8	14	
	**EC**	5	7	
	**TX**	14	10	

*Abbreviation*: *HR* Hormone receptor, *Her-2* Human epidermal growth factor receptor-2, *TNBC* Triple negative breast cancer, *NACT* Neoadjuvant chemotherapy, *CR* Chemical regime, *TEC* Docetaxel + anthracyclines + cyclophosphamide, *EC* Anthracyclines + cyclophosphamide, *TC* Docetaxel + cyclophosphamide, *DP* Docetaxel + platinum

### The value of TIN’s change before and after NACT for chemotherapy efficacy and DFS in patients with breast cancer

In this study, low difference value of TIN was associated with high rates of recurrence and low rates of remission (shown in Fig. 2b and Supplement Fig. 2, b). In Supplement Table 1, the value of TIN’s change was identified as a significant predictor for DFS (*p* = 0.002, hazard ratio [HR] = 0.984; 95% [CI] 0.974–0.994 in univariate analysis). In multivariate analysis, the value of TIN’s change was significantly associated with the rate of recurrence (*p* = 0.003, hazard ratio [HR] = 0.984; 95% [CI] 0.973–0.994), and the C-index of TIN’s change (0.64, 95%CI 0.63–0.65) was higher than that of TIN after NACT (0.62, 95% [CI] 0.61–0.63).

## Discussion

In this study, patients with higher TIN after NACT were more inclined to suffer resistance to NACT and recurrence. In addition, this is the first study to demonstrate that the TIN’s dynamic change before and after NACT was significantly associated with better chemotherapy efficacy and prognosis in patients with breast cancer.

Previous researches have confirmed the prognostic role of tumor infiltrating lymphocyte (TIL) in breast cancer patients [27–31]. As an indispensable component of tumor immune cells, TIN was reported to have a significant impact on TME [32]. Wang J et al. [33] had implicated TIN as an independent marker of prognosis in biliary cancer patients. Jensen HK et al. [18] revealed that presence of TIN was an independent prognostic factor in localized renal cell carcinoma. When concerning breast cancer, studies have revealed that TIN may be associated with metastasis [16, 34]. After stratifying patients into two groups according to the optimal cut-off value of TIN after NACT for chemotherapy efficacy, in accordance with previous studies, the subgroup with lower TIN (< 52.2) was associated with longer DFS. In addition, patients with higher TIN after NACT were more inclined to be resistant to NACT. The reason may be that the higher concentration of TIN after NACT correlates with the inhibition of anti-tumor immunity and the initiation of metastasis [16, 34]. However, the specific mechanism accounting for the association remains unclear.

The change of composition in TME may reflect the TME’s response to NACT. Given that the status of TIN after NACT could be associated with clinical outcome of breast cancer patients, we concentrated further on the significance of dynamic change of TIN before and after NACT. In this study, we revealed that it was significantly associated with chemotherapy efficacy (shown in Table 2, *p* < 0.001) and prognosis of breast cancer patients (*p* = 0.002, hazard ratio [HR] = 0.984; 95% [CI] 0.974–0.994 in univariate analysis and *p* = 0.003, hazard ratio [HR] = 0.984; 95% [CI] 0.973–0.994 in multivariate analysis). Our study showed that the dynamic change of TIN could better predict the chemotherapy efficacy and prognosis than the stationary state of TIN after NACT. The reason may be that the dynamic change of TIN partially reflected the response of TME to NACT, which indicated whether pro-tumor inflammation or anti-tumor immune played a major role in TME. For the high difference group patients, the reduction of TIN may reduce the impact on anti-tumor immune response, leading to better immune killing effect conducted by TIL, and favorable chemotherapy efficacy. Therefore, it may serve as a marker for evaluating the curative effect and predicting prognosis in patients with breast cancer patients receiving NACT, and may provide a new insight for immunotherapy of breast cancer in the future.

Some limitations in the present study need to be considered. First, the number of patients enrolled in this study was relatively small. In order to better evaluate and analyze the correlation between TIN and the chemotherapy efficacy and prognosis of breast cancer patients, more patients need to be enrolled in the study from multiple centers to verify the results using a validation cohort and to improve the credibility of the study. Second, our study didn’t explore the mechanism of TIN in TME. Therefore, these results still need a prospective, large, multicentered randomized trial to validate.

## Conclusion

TIN after NACT was identified as an independent prognostic factor for breast cancer patients. Furthermore, we revealed that the difference of TIN before and after NACT may become a new, strong, independent prognostic factor for clinical outcome of patients with breast cancer.

## Supplementary Information

**Additional file 1: Supplement Figure 1.** Chemotherapy regime in low and high difference group.

**Additional file 2: Supplement Figure 2.** Analysis of patients achieving remission after NACT in high and low TIN groups (a), in low and high difference group (b). TIN = tumor infiltrating neutrophil.

**Additional file 3: Supplement Figure 3.** Representative microphotographs of CD66b staining. Tumor tissue with no CD66b staining (a). Tumor tissue with weak CD66b staining (b). Tumor tissue with CD66b strong staining (c), (magnification 200×).

**Additional file 4: Supplement Figure 4.** Representative microphotographs of CD66b staining before and after NACT. CD66b staining before NACT (a, c) and CD66b staining after NACT (b, d). Positive staining was brown and magnification was 200×. The representative staining a and b were from the same patient (low difference group). C and d were from another patient (high difference group).

**Additional file 5: Supplement Table 1.** Clinicopathological Characteristics: Univariate and Multivariate Survival Analyses.

## Data Availability

The dataset of the current study was available from the corresponding author on reasonable request.

## Abbreviations

TIN: Tumor infiltrating neutrophil
BCS: Breast-conserving surgery
HR: Hormone receptor
Her-2: Human epidermal growth factor receptor-2
TNBC: Triple negative breast cancer
DFS: Disease-free survival
NACT: Neoadjuvant chemotherapy
PCR: Pathological complete response
CR: Chemical regime
TEC: Docetaxel + anthracyclines + cyclophosphamide
EC: Anthracyclines + cyclophosphamide
TC: Docetaxel + cyclophosphamide
DP: Docetaxel + platinum
TME: Tumor microenvironment
