# Supplementary figures and images for: Tumor infiltrating neutrophil might play a major role in predicting the clinical outcome of breast cancer patients treated with neoadjuvant chemotherapy
Source: BMC Cancer. 2021 Jan 14;21:68. doi: 10.1186/s12885-021-07789-6 (PMC7809871; doi:10.1186/s12885-021-07789-6)

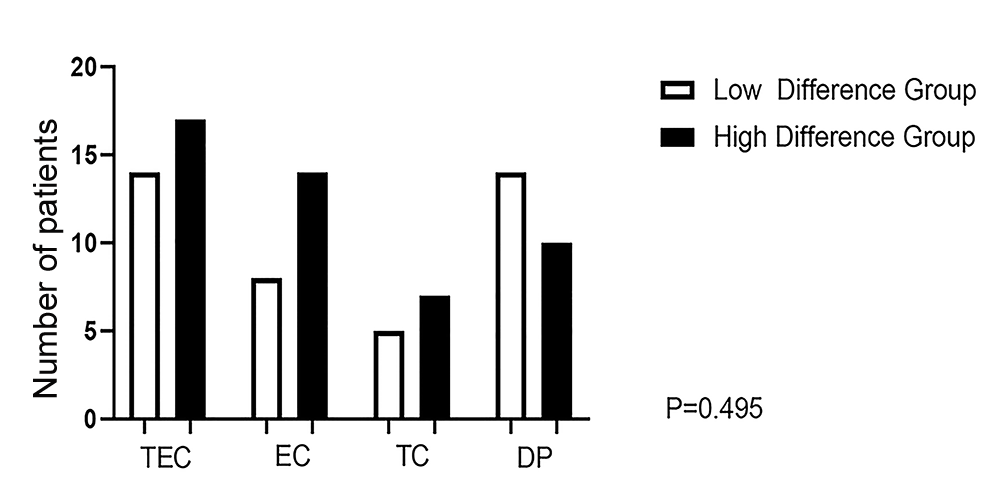

Supplement: Supplementary file 1 — Additional file 1: Supplement Figure 1. Chemotherapy regime in low and high difference group. [file 12885_2021_7789_MOESM1_ESM.tif]

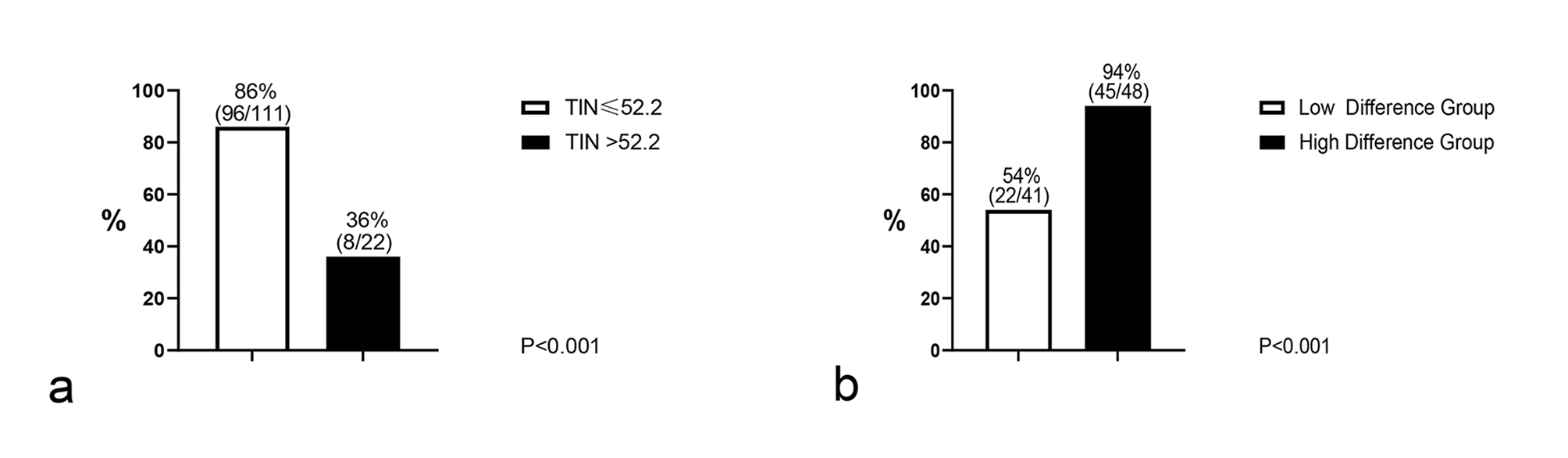

Supplement: Supplementary file 2 — Additional file 2: Supplement Figure 2. Analysis of patients achieving remission after NACT in high and low TIN groups (a), in low and high difference group (b). TIN = tumor infiltrating neutrophil. [file 12885_2021_7789_MOESM2_ESM.tif]

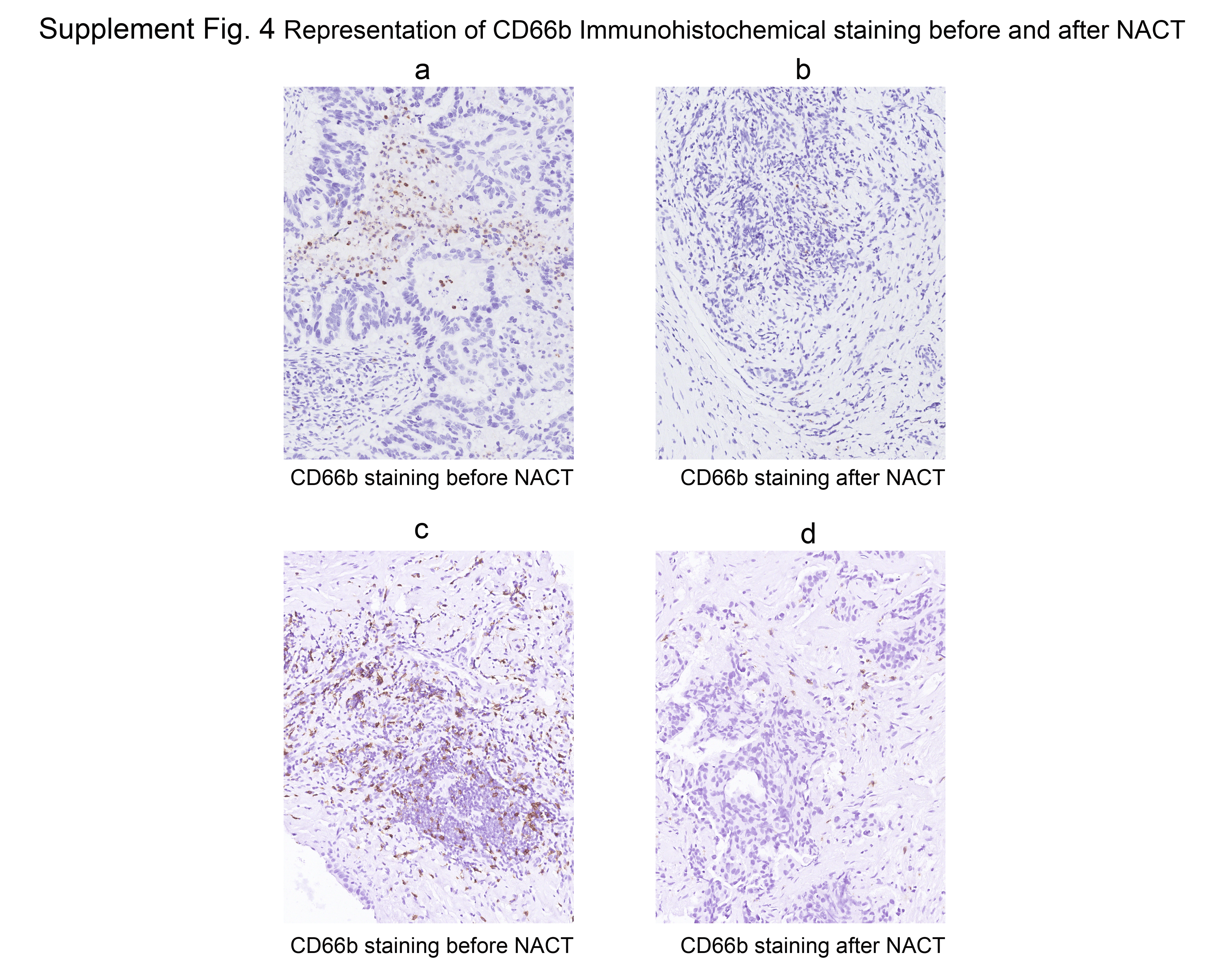

Supplement: Supplementary file 4 — Additional file 4: Supplement Figure 4. Representative microphotographs of CD66b staining before and after NACT. CD66b staining before NACT (a, c) and CD66b staining after NACT (b, d). Positive staining was brown and magnification was 200×. The representative staining a and b were from the same patient (low difference group). C and d were from another patient (high difference group). [file 12885_2021_7789_MOESM4_ESM.tif]
