# Supplementary material for: Tumor infiltrating neutrophil might play a major role in predicting the clinical outcome of breast cancer patients treated with neoadjuvant chemotherapy
Source: BMC Cancer. 2021 Jan 14;21:68. doi: 10.1186/s12885-021-07789-6 (PMC7809871; doi:10.1186/s12885-021-07789-6)
Supplement: Supplementary file 5 — Additional file 5: Supplement Table 1. Clinicopathological Characteristics: Univariate and Multivariate Survival Analyses. [file 12885_2021_7789_MOESM5_ESM.docx]

| **Supplement Table 1. Clinicopathological Characteristics: Univariate and Multivariate Survival Analyses** | | | | | |
| --- | --- | --- | --- | --- | --- |
| **Characteristic** | **DFS** | | | | |
|  | **Univariate** | | **Multivariate** | | |
|  | **HR (95%CI)** | **P** | | **HR (95%CI)** | **P** |
| **Age** | 1.033 (0.995-1.072) | **0.007** | | 1.031 (0.953-1.117) | 0.444 |
| **Menopause vs**  **no menopause** | 1.693 (0.672-4.268) | 0.257 | | 0.934 (0.143-6.118) | 0.944 |
| **Stage**  **(III vs II vs I)** | 4.411 (1.396-13.938) | **0.015** | | 3.465 (1.050-11.429) | **0.041** |
| **Molecular type**  **(HR+ vs Her-2 vs TNBC)** | 0.926 (0.559-1.534) | 0.756 | | 0.905 (0.538-1.520) | 0.705 |
| **The value of TIN’s change** | 0.984 (0.974-0.994) | **0.002** | | 0.984 (0.973-0.994) | **0.003** |

Abbreviation: HR = hormone receptor; Her-2 = Human epidermal growth factor receptor-2; TNBC = triple negative breast cancer;

DFS = disease free survival; TIN = tumor infiltrating neutrophil
